# Organic Selenium as Antioxidant Additive in Mitigating Acrylamide in Coffee Beans Roasted via Conventional and Superheated Steam

**DOI:** 10.3390/foods9091197

**Published:** 2020-08-29

**Authors:** Ahmad K. Alafeef, Fazilah Ariffin, Musfirah Zulkurnain

**Affiliations:** Food Technology Division, School of Industrial Technology, Universiti Sains Malaysia, 11800 Minden, Penang, Malaysia; afeef@student.usm.my (A.K.A.); musfirah.z@usm.my (M.Z.)

**Keywords:** selenomethionine, green coffee, acrylamide, Arabica, Robusta, Maillard reaction, selenium uptake, pretreatment

## Abstract

Selenium is an essential micronutrient with significant antioxidant activity promising in mitigating the formation of acrylamide during high-temperature roasting. In this study, green coffee beans pretreated with selenium (Se-coffee) were investigated on their selenium uptake, selenium retention in green and roasted beans, antioxidant activities, and formation of acrylamide during conventional and superheated steam roasting. Comparisons were made with positive (pretreated without selenium) and negative (untreated) controls. The acrylamide formation was significantly inhibited in Se-coffee (108.9–165.3 μg/kg) compared to the positive and negative controls by 73.9% and 52.8%, respectively. The reduction of acrylamide by superheated steam roasting only observed in the untreated coffee beans (negative control) by 32.4% parallel to the increase in its antioxidant activity. Selenium pretreatment significantly increased antioxidant activity of the roasted Se-coffee beans after roasting although soaking pretreatment significantly reduced antioxidant activity in the green beans. Acrylamide reduction in the roasted coffee beans strongly correlated with the change in antioxidant capacities after roasting (∆FRAP, 0.858; ∆DPPH, 0.836). The results indicate that the antioxidant properties of the organic selenium suppressed acrylamide formation during coffee roasting.

## 1. Introduction

Coffee is consumed widely around the world, mainly due to its refreshing and stimulating effects. Arabica (*Coffea arabica*) and Robusta (*Coffea canephora*) are two coffee species most widely cultivated with the former known for better organoleptic characteristics, while Robusta coffee possesses higher antioxidant activity but less favorable flavor. The potential benefits of coffee consumption have been well studied to associate with its rich phytochemicals and antioxidant properties originating from the green beans and complex bioactive compounds formed during coffee roasting [1]. Main bioactive compounds in coffee brew are chlorogenic acids, caffeine, pentacyclic diterpenes (cafestol and kahweol), trigonelline, and melanoidins which have been associated with reduced incidences of developing neurodegenerative diseases, several types of cancer, cardiovascular diseases, and type 2 diabetes [2,3]. Antioxidative properties of coffee brews are attributed to phenolic compounds predominantly from melanoidins, chlorogenic acids, and caffeine [4,5,6]. The contribution of coffee to the daily intake of dietary antioxidants for many people is more than other food sources such as fruit, vegetables, and herbs [7,8].

Roasting of coffee beans at high temperatures involves a series of reactions including caramelization, Maillard reaction, Strecker degradation, and pyrolytic reaction responsible for the development of desirable organoleptic characteristics and antioxidant capacity of coffee [9,10]. Maillard reaction products (MRP) formed during roasting are important flavor and color components in roasted coffee, chiefly Melanoidins, strong antioxidants that account for ~29% of coffee brew dry matter [6]. However, the Maillard reaction also contributes to the formation of undesired toxic components that may counteract the health benefits of coffee, such as acrylamide. Acrylamide is classified as carcinogenic compounds for human health (Group 2A) due to neural, reproductive, and genetic toxicities [11]. Roasted coffee contributes to significant levels of dietary exposure of acrylamide, leading to the recent establishment of the benchmark level of acrylamide in roasted ground coffee at 400 μg/kg by the European Commission [12].

Acrylamide forms in coffee during roasting via reaction of carbonyl group mainly from degradation products of sugars and polysaccharides with amino group of asparagine in Maillard reaction. Mitigation of acrylamide during coffee roasting should target for limiting the source of carbonyl and asparagine content of the coffee beans. Several studies have demonstrated the ability of antioxidants to inhibit the formation of acrylamide in several food matrixes by limiting the source of carbonyl pool, reacting with key Maillard reaction intermediates (e.g., 3-aminopropionamide), and with the acrylamide itself [13]. However, some antioxidants showed contradictory observations by increasing acrylamide levels. Different structures and functional groups of antioxidants can induce the formation of reactive carbonyl pool at high temperatures and low moisture conditions [13]. In particular, antioxidants that contain carbonyl groups such as chlorogenic acids and caffeine were shown to accelerate the formation of acrylamide [14,15,16]. Studies using several model-systems simulating coffee showed that chlorogenic acid triggered the decomposition of sugars to form 3,4-dideoxyosone and 5-hydroxymethylfurfural (HMF), which then able to react with asparagine via Maillard reaction and promote the formation of acrylamide [14,15,16]. Alternatively, organic selenium, such as L(+)-selenomethionine, has a high melting properties above 250 °C and remains stable at high temperatures [17]. Therefore, it can be utilized as an antioxidant additive and supplemented to green beans for reducing the formation of acrylamide during coffee roasting.

Selenium is essential to human health with a recommended daily allowance (RDA) value of 55 μg/day and a tolerable upper intake of 400 μg/day for adults [18]. Selenium plays a role in several major metabolic pathways such as immune functions, antioxidants defense systems, and thyroid hormone metabolism [19]. Simultaneously, the deficiency of selenium is associated with cardiovascular and inflammatory diseases, cancer, cirrhosis, diabetes, asthma, and other free radical related problems such as premature aging. Recently, there has been intense interest in selenium supplementation due to its role in protecting the immune system, improve cardiovascular functions, and protection against cancer [20,21]. Coffee contains a trace amount of selenium in the organic form, which is not significant for selenium supplementation [22]. The infusion of selenium via raw material pretreatment has been shown to contribute to aroma profile of roasted coffee and enrichment of its natural selenium level [23] but no impact on the fate of acrylamide is available.

The mitigation strategies of acrylamide in coffee have taken multiple approaches from the modification of raw material to remove potential precursors (e.g., steam and enzyme pretreatments of green beans) and modification of roasting process (e.g., vacuum and steam roasting) to the addition of external additives (e.g., adding amino acid) [24,25,26,27]. However, the impact on the sensorial and nutritional properties of coffee are among the limitations of the intervention studies. Superheated steam roasting demonstrated improvement in sensorial quality, increase antioxidant capacity, and reduce lipid oxidation of roasted coffee beans due to the generation of oxygen absent environment at higher bed temperature [9,28,29]. Nevertheless, no information is available on the effect of superheated steam roasting on the fate of acrylamide in coffee. The present study aimed to investigate the effects of selenium pretreatment on the acrylamide formation in Arabica and Robusta coffee during roasting in relation to their antioxidant activities, comparing the conventional and superheated steam roasting. The positive and negative controls were employed to evaluate the true effects of organic selenium on acrylamide formation in the coffee beans during roasting.

## 2. Materials and Methods

### 2.1. Materials

Single-origin green Arabica (Santos, Brazil) and Robusta (Laos) coffees beans were obtained from Rasta Brew Ent. (Penang, Malaysia). Selenomethionine, acrylamide (99.3% purity), and 2,2-diphenyl-1-picrylhydrazyl (DPPH) were supplied by Sigma Aldrich (St. Louis, MO, USA).

### 2.2. Selenium Pretreatment

The green beans were cut using laboratory mills (ZM 200, Retsch GmbH, Haan, Germany) to one third in size to increase surface area for selenium uptake. The crushed green beans (100 g) were soaked overnight in different concentrations of selenomethionine solutions (0, 200, 400 μg/L) at the ratio of 1:5 (*w*/*v*) at 25 °C. The Se-coffee beans were then subjected to drying using the hot air dryer at 45 °C until moisture content reached ~10% prior to roasting. The selenium pretreatment was investigated by comparing it with a positive and negative control sample. The positive control was the sample pretreated with similar procedures in the absence of selenium, while the negative control sample was the untreated green coffee beans.

### 2.3. Coffee Roasting

The coffee roasting was done in a laboratory drum roaster hosted in a superheated steam oven (Healsio, AV-1500V, Sharp, Japan). The perforated metal drum is equipped with a motor (Buiacs, JWD Motor Co., Ltd., Zhejiang, China) to spin the drum, short fins at the sides of the drums to allow the beans to fall to the center of the drum, and thermocouple at the center of the drum for actual temperature monitoring. The oven is functional for both conventional roasting and roasting with superheated steam at a pressure of approximately 1 bar, steam generation capacity of 16 cm^3^/min, and steam engine heater of 900 W. The oven was preheated to 240 °C before roasting 100 g green beans for 20 min. The roasted coffee beans were then cooled to room temperature and stored in the air-tight container for subsequent analyses.

### 2.4. Determination of Acrylamide in Coffee

The acrylamide determination was conducted according to PerkinElmer (2004) and Ku Madihah et al. (2013) [30,31]. Ground roasted coffee beans (5 g) was mixed with 50 mL distilled water and heated at 50 °C under continuous stirring for 10 min. The mixture was filtered using filter paper (No. 4, Whatman, Darmstadt, Germany) and loaded to SPE Bond Elut C18 (Agilent, Santa Clara, CA, USA) under gravity flow. The SPE cartridge (500 mg) was conditioned with 3 mL acetone followed by 3 mL 0.1% formic acid. After extraction, the SPE cartridge was washed with 2 mL deionized water and applied vacuum to remove excess water. The acrylamide was eluted using 3 mL of acetone and passed through a nylon syringe filter (0.45 μm, Whatman, Darmstadt, Germany). The sample was kept at 4 °C until further GC-FID analysis.

Acrylamide was determined using a gas chromatograph with a flame ionization detector (Shimadzu Corp, Kyoto, Japan) using the Elite-Wax ETR column (15 m, 0.53 ID, 0.50 µm film thickness) (PerkinElmer Inc., Waltham, MA, USA). Working conditions were as follows: Carrier gas helium (1 mL/min at constant flow); injector, 260 °C; oven temperature: From 100 °C (0.5 min) to 200 °C at 15 °C/min.

Quantification of acrylamide was carried out by preparing a standard curve (r^2^ = 0.996) with eight different concentration levels of acrylamide (0.02–2000 µg). Data are mean values of at least two experiments with coefficients of variation at the different concentrations lower than 10%.

### 2.5. Determination of Selenium

The selenium determination was conducted according to Choi et al. (2009) with slight modifications [32]. An amount of 5 g of finely ground coffee samples was weighed accurately and digested with 40 mL of nitric acid in a digestion tube for 4 h at 150 °C until the darkness of the solution disappeared. After cooling, the digested solution was diluted with deionized water and transferred to a 50 mL volumetric flask. The diluted solution was filtered through filter paper (No. 4, Whatman, Darmstadt, Germany) into a plastic tube designed for the autosampler analysis. Total selenium levels were measured in the aqueous selenium solutions before and after the beans were removed. The difference in selenium levels correspond to the selenium uptake by the coffee beans (µg/kg) [23].

Selenium was determined using NexION™ 300Q Perkin Elmer ICP–MS (PerkinElmer Inc., Waltham, MA, USA) with analysis conditions as follows: Forward power, 1000 W; plasma gas flow rate (Ar), 17.0 L/min; auxiliary gas flow rate (Ar), 1.20 L/min; nebulizer gas flow rate (Ar), 0.93 L/min; Baffled Cycronic Spray Chamber; Ni/Ni sampling cone/skimmer cone; dwell time, 0.50 s per isotope; isotope monitored, Se^82^. Selenium concentrations of the samples were quantified using the selenium standard curve (r^2^ = 0.999).

### 2.6. Determination of Antioxidants Activity

The ground green and roasted coffee beans (1 g) were extracted with 40 mL of methanol for two hours and the extract was filtered using filter paper (No. 1, Whatman, Darmstadt, Germany).

#### 2.6.1. FRAP Assay

The FRAP values were determined according to Moreira et al. (2005) with slight modification [33]. The FRAP reagent was prepared by adding a 10 mM tripyridyl triazine (TPTZ) solution in 40 mM HCl and 20 mM of FeCl_3_. 6H_2_O to 300 mM sodium acetate buffer (pH 3.6) in a ratio 1:1:10 (*v*/*v*/*v*) and incubated in a water bath at 37 °C for 30 min. An amount of 100 μL of each sample extract was mixed with 900 μL FRAP reagent. The mixture was incubated for 30 min at 37 °C and the absorbance at 593 nm was recorded using an ultraviolet-visible spectrophotometer (UV-1650PC, Shimadzu Corp., Kyoto, Japan). The FRAP values were obtained compared to the calibration curve for Fe^+2^ (r^2^ = 0.998), expressed as mmol of Fe^2+^ equivalents per gram of soluble solids.

#### 2.6.2. DPPH Assay

The DPPH assay for antioxidant activity was conducted according to Kwak et al. (2017) [34]. A methanol solution of DPPH (2,2-diphenyl-1-picrylhydrazyl) (50 mg/100 mL) at 0.2 mL was mixed with the extract, and the mixture was brought to a total volume of 4.0 mL. The mixture was mixed and kept in the dark for 45 min. Absorbance reading was taken at 515 nm (A_S_), along with a blank prepared by substituting the coffee extract with methanol (A_c_). DPPH radical scavenging activity was calculated using the following equation:

DPPH radical scavenging ratio (%) = ((A_c_ − A_S_)/A_c_) × 100 (1)

The concentration of samples required to reduce the absorbance of DPPH by 50% (EC_50_) expressed as mg/mL was determined from the regression of the curve of the methanolic solution of the coffee extract in five different concentrations.

### 2.7. Color Evaluation

Color measurements of ground roasted coffee were carried out using the CIE Lab-scale spectrophotometer (CM-3500D, Minolta Co., Ltd., Osaka, Japan). The measurement time was set at 2.5 s, the reflectance used was d/8 (diffuse illumination/viewing angle) geometry, and pulsed xenon arc lamp was used the light source. The color values of the ground roasted coffee were expressed as L* (luminosity component), a* (redness/greenness), and b* (blueness/yellowness). The browning index (BI) was calculated using Equations (2) and (3) to estimate the purity of brown color as an indicator of the extend of non-enzymatic browning reaction according to Virgen-Navarro et al. (2016) and Buera et al. (1986) [35,36].

Browning index, BI = (z − 0.31)/(0.172 × 100),(2)

where

Chromaticity coordinate of a color, z = (a* + 1.75L*)/(5.645L* + a* − 3.012b*) (3)

### 2.8. Statistical and Data Analysis

All data expressed as means ± standard deviation were analyzed using MINITAB statistical software version 19 (Minitab, Inc., State College, PA, USA) for the one-way analysis of variance (ANOVA) with post-hoc Tukey’s tests and Pearson correlation at a 0.05 significance level. Furthermore, two-way ANOVA was applied to determine the significant effects of linear as well as interaction of independent variables. A significance level of *p* < 0.05 was used throughout the study. All measures were taken in triplicate.

## 3. Results and Discussion

### 3.1. Selenium Uptake by Green Coffee Beans and Retention in Se-Coffee Beans

**Figure 1:**
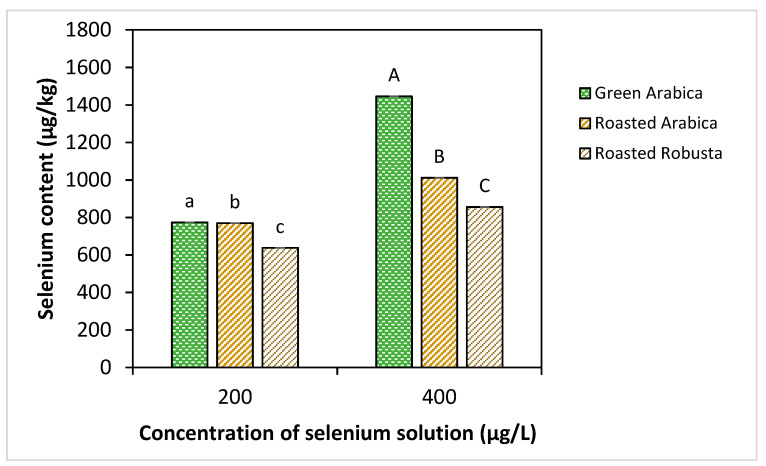
Selenium content of green Arabica, and roasted Arabica and Robusta Se-coffee beans (μg/kg) pretreated at different selenium concentrations and roasted via conventional roasting. Lowercase and uppercase letters represent significant differences (*p* < 0.05) between samples at low and high selenium concentrations, respectively.

Figure 1 shows the selenium retention in the green and roasted Se-coffee beans pretreated at high and low selenium concentrations. The selenium uptake by the crushed Arabica green beans after overnight soaking was at ~71% and 77% from the 200 and 400 μg/L selenium solutions, respectively. An increase in selenium concentration significantly increased selenium uptake by the green beans as a higher concentration gradient is expected to increase the rate of selenium diffusion into the beans. The actual selenium content in the green Se-coffee beans was determined after the drying process found at 774.0 and 1446.0 μg/kg for low and high selenium concentrations, respectively. The loss of selenium after drying process was at ~46 and 53% for low and high selenium concentrations, respectively.

Roasting of the Arabica and Robusta Se-coffee beans showed no significant loss of selenium at lower selenium concentration with a final selenium content of 770 μg/kg in Arabica Se-coffee bean. For higher selenium concentration, ~30% selenium loss was observed after roasting with final selenium content at 1012.0 μg/kg. The loss of selenium was higher since the rate of chemical reactions depends on the concentration of reactants. Volatile selenium species have been reported in roasted coffee beans pretreated with selenomethionine and selenocysteine at a higher concentration (10 mg/L) that may suggest the loss of selenium during coffee roasting [23]. Between different coffee varieties, Robusta coffee showed significantly lower final selenium content after roasting at 638.0 and 855.8 μg/kg for both low and high selenium concentrations, respectively.

The Se-coffee pretreated with high selenium concentration can contribute to selenium content at about 13.6 and 11.5 μg per serving from “double shot” espresso (20 g ground beans) of Arabica and Robusta varieties assuming 67% recovery which equivalent to 24.7 and 20.8% of selenium RDA, respectively [37]. The possibility of exceeding the UL of selenium (400 μg/day) at 30 cups of “double shot” espresso is very unlikely. Thus, the higher selenium concentration was selected for the acrylamide mitigation study.

### 3.2. Effects of Selenium Pretreatment on Acrylamide Formation

**Figure 2:**
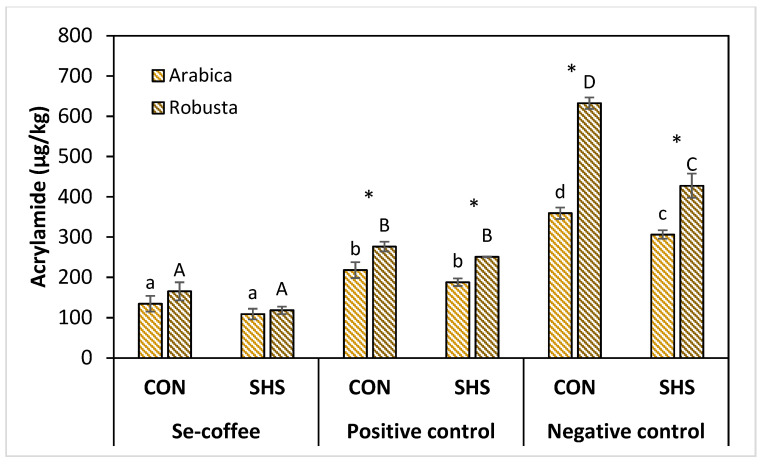
Acrylamide levels in Arabica and Robusta Se-coffee (pretreated with selenium), positive control (pretreated without selenium), and negative control (untreated) roasted via conventional (CON) and superheated steam (SHS) roasting. Lowercase letter, uppercase letter, and star symbol represent significant differences (*p* < 0.05) between different treatments for Arabica, Robusta coffees, and significant differences (*p* < 0.05) between the coffee varieties, respectively.

Figure 2 shows the acrylamide levels in Se-coffee compared to the positive control (pretreated without selenium) and negative control (untreated) roasted via conventional and superheated steam roasting for Arabica and Robusta. The two-way ANOVA of the data showed stronger influence of the selenium pretreatment on the acrylamide formation (*F*-value = 54.61; *p*-value = 0.000, Figure 2) compared to the roasting method (*F*-value = 7.31; *p*-value = 0.011, Figure 2) with insignificant interaction effect between them. The selenium pretreatment significantly (*p* < 0.05) reduced the acrylamide levels in Se-coffee (108.9–134.4 μg/kg) compared to the negative control (306.2–359.2 μg/kg) by ~64% in Arabica coffee. Robusta coffee showed higher acrylamide levels at 118.4–165.3 μg/kg and significantly (*p* < 0.05) larger acrylamide reduction compared to the negative control (427.3–632.4 μg/kg) at ~73%.

The pretreatment alone without selenium (positive control) significantly (*p* < 0.05) reduced acrylamide levels to 187.7–217.9 μg/kg and 250.8–276.3 μg/kg for Arabica and Robusta coffee, respectively with ~39 and 40% reduction compared to the negative controls. The pretreatment involving overnight soaking of coffee beans in water could facilitate the leaching of water-soluble precursors of Maillard reaction. Soaking green coffee beans in the wet processing method of coffee production has reported the loss of sugars (sucrose, glucose, fructose, mannitol), amino acids (asparagine, arginine, and aspartic acid), chlorogenic acids, and alkaloids (trigonelline and caffeine) through leaching [38]. The concentrations of sucrose, raffinose, and stachyose reduced to half after 40 h soaking, with a higher final concentration of sugars reported in Robusta coffee [39], which may elucidate the higher acrylamide levels of Robusta compared to Arabica coffee in the positive and negative controls.

Selenium supplementation resulted in about two-fold acrylamide reduction (~40–52.8%) compared to the pretreatment alone. These results suggest the role of selenium as an antioxidant additive in suppressing acrylamide formation during coffee roasting by reacting further with the precursors of Maillard reaction and Maillard reaction intermediates. Organic selenium can participate in Maillard reaction to form organoselenium of mono-, di-, and triselenium compounds such as methyl selenoacetate, 2- and 3-methylfuranyl methyl selenide, dimethyl diselenide, and 1,2,4-selenotrithiolane studied in a selenomethionine-glucose model system [40]. Large part of the volatile organoselenium generated is dimethyl diselenide, demonstrating a higher reactivity towards the Maillard reaction than selenomethionine [41].

Roasting methods showed significant influence on the acrylamide formation only in the untreated coffee beans (negative control). The superheated steam roasting significantly (*p* < 0.05) reduced acrylamide formation by 14.8% from 359.2 to 306.2 μg/kg for Arabica coffee and at 32.4% from 632.4 to 427.3 μg/kg for Robusta coffee, respectively. These results suggest that chemical pretreatment is more effective than physical treatment in mitigating acrylamide in coffee, attributing to the removal of associated precursors. The lack of oxygen condition is the most important characteristic of the superheated steam roaster, because the air in the system is replaced by superheated steam, thus, the sample heated under environment lack of oxygen is not oxidized [9,28]. It has been reported that the coffee beans roasted under superheated steam had lower pH and higher sugar content [9,29]. The superior thermal properties of superheated steam to hot air have been reported to result in the lower formation of sugar degradation products in coffee which can limit the formation of Maillard reaction intermediates and formation of acrylamide [9].

Furthermore, the superheated steam roasting has been shown to reduce lipid oxidation under the environment of very small amount of oxygen (~2–3%) during roasting of high lipid products [42,43]. Roasting coffee under superheated steam has been reported to decrease the unsaturated aldehydes content generated during roasting from lipid oxidation reaction [29]. Several researchers suggested the role of lipid oxidation products contributing to carbonyls pool at a significant amount during coffee roasting as an alternative route for acrylamide formation [14,44,45]. Linoleic acid as predominant lipid in coffee beans is likely to oxidize under roasting conditions into corresponding esters containing reactive α,β,γ,δ-diunsaturated carbonyl group which shown increased reactivity by 1.6 fold towards asparagine in the presence of sugar in a binary system [14,45,46]. In the untreated green coffee beans, lipid (8–18%) and soluble carbohydrates (6–12%) of mainly sucrose occur at almost equal concentration, thus the synergistic effect is highly likely to result in high acrylamide formation in the conventional roasting setting in which the lipid oxidation is the preliminary step [45,46]. The significant acrylamide reduction in the superheated steam roasted coffee may be attributed to the lower Maillard reaction precursors from sugar and lipid oxidation products due to lack of oxygen under superheated steam conditions [47]. Results in this study suggest that superheated steam roasting can be an effective physical method to mitigate acrylamide.

### 3.3. Effects of Selenium Pretreatment on Antioxidant Activity of Green and Roasted Coffee Beans

#### 3.3.1. Green Beans

The antioxidant activity of green coffee beans is attributed to their complex bioactive constituents mainly caffeine and chlorogenic acid of hydrophilic nature and trigonelline, cafestol, and kahweol of hydrophobic nature [4,5]. Because of different chemical and physical characteristics of different antioxidants and radical sources, no single assay will accurately reflect antioxidant activity in a complex matrix like coffee [48]. The antioxidant activity of green coffee beans is widely measured by means of ferric reducing antioxidant power (FRAP) assay based on the reduction of transition metal ions by hydrophilic antioxidants and 2,2-diphenyl-1-picrylhydrazyl (DPPH) assay of stable lipophilic radical based on radical quenching which also permits the determination of hydrophobic antioxidants [5,49]. The DPPH radical scavenging activity of the coffee extract is represented as an effective concentration necessary to reduce 50% of the DPPH radicals (EC_50_) [5]. Two major reaction mechanisms of antioxidant with free radicals are hydrogen atom transfer (HAT) or single electron transfer (SET), or the combination of both mechanisms [48]. FRAP is a SET based assay that evaluate the participation of single electron transfer from nucleophile to substrate to produce radical cation that has the ability to stops radical chains [48]. However, FRAP cannot detect compounds with free radical scavenging activity. Thus, DPPH that represents both HAT and SET reaction mechanisms, can be used in combination with FRAP to distinguish different reaction mechanisms of antioxidants in coffee. The antioxidant activities of different organic and inorganic selenium species have been reported by Sentkowska and Pyrzyńska (2019) with selenomethionine showing highest antioxidant activities measured using DPPH, cupric reducing antioxidant capacity (CUPRAC) and Folin–Ciocalteu (FC) assays [50]. However, the author reported reduction in their radical scavenging activities when mixed with tea extracts due to alteration in antioxidant reaction mechanism in the presence of active components such as phenolic compounds. Therefore, in this study the comparison of antioxidant activities of the Se-coffee was not made with the pure selenomethionine, rather with coffee samples of different pretreatments.

The antioxidant activities of green Se-coffee, positive, and negative controls measured by FRAP and DPPH EC_50_ values are shown in Table 1. The FRAP values for untreated green coffee beans (negative control) were at 1.86 and 2.07 mmol Fe^2+^ eq/g for Arabica and Robusta coffees, respectively. The pretreatments involving soaking significantly (*p* < 0.05) reduced FRAP values of the green Se-coffee (0.58 mmol Fe^2+^ eq/g) and the positive control (0.47 mmol Fe^2+^ eq/g) by ~74 and 69%, respectively compared to the untreated Arabica beans. For the Robusta beans, the pretreatments resulted in a lower reduction of FRAP value with Se-coffee (1.00 mmol Fe^2+^ eq/g) significantly higher than the positive control (0.84 mmol Fe^2+^ eq/g).

The radical scavenging activity of the green beans was at 1.93 and 1.92 mg/mL for Arabica and Robusta coffees, respectively. The lower amount required to give EC_50_ indicates higher radical scavenging activity. The pretreatments resulted in an increment of EC_50_ values of the Se-coffee (3.19 mg/mL) and the positive control (3.38 mg/mL) by 65 and 75%, respectively. Robusta coffee with higher antioxidant capacity also showed comparable trends at 67 and 64% increment for the Se-coffee (3.20 mg/mL) and the positive control (3.15 mg/mL). The selenium supplementation showed no significant decrease in the EC_50_ values of Se-coffee compared to the positive control samples. These observations are in agreement with the previous finding, which observed no significant effects of selenium infusion on the radical scavenging activity of black and green tea extracts [50]. This observation may suggest the formation of complex interactions between selenomethionine and other antioxidant and phenolic compounds in the green coffee beans with different antioxidant activity.

Polyphenols compounds of predominantly chlorogenic, ferulic, and caffeic acids account for 6–10% of green coffee bean’s dry weight may largely contribute to the total antioxidant activity of untreated green beans. However, soaking of the crushed green coffee beans overnight is expected to result in leaching of water-soluble constituents with antioxidant properties into the soaking mediums. Several studies on extra soaking step in wet processing method of coffee beans reported leaching of soluble portion of the green beans, including diterpenes, polyphenols, and tannins with antioxidant properties such as caffeine, trigonelline and chlorogenic acids from the green coffee beans [38,51]. The loss of chlorogenic acids from green coffee beans was reported up to 50% of caffeoylquinic acids (CQAs) notably the 3,4-diCQA and 3,5-diCQA isomers due to soaking and degradation during drying [38]. In this study, further breaking of the beans and drying can expect a higher loss of these antioxidants from the pretreated samples. The selenium pretreatment significantly increased the antioxidant activity of Se-coffee by means of FRAP values but not EC_50_ because the FRAP assay measures the total antioxidant activity of the sample, representing the combined single-electron transfer reductive ability of all redox-active antioxidants present in the samples compared to the DPPH assay [52].

#### 3.3.2. Roasted Beans

Roasting coffee at high temperatures results in changes in coffee compositions mainly on the degradation of polyphenol components and the formation of Millard reaction products that contributes to changes in its final antioxidant activities [53]. The FRAP and DPPH EC_50_ values of roasted coffee beans as affected by the selenium pretreatment and roasting methods are shown in Table 2. It is expected that the antioxidant activity of the negative control (untreated samples) significantly (*p* < 0.05) reduced after roasting. This is especially true for dark roasting degree as the thermal degradation of polyphenols is not counterbalanced by the formation of non-phenolic Millard reaction products such as melanoidins [54,55]. Interestingly, the antioxidant activities of the roasted Se-coffee and positive control were comparable to the negative control with the Se-coffee showed higher values for both antioxidant assays. Roasting of the Se-coffee significantly (*p* < 0.05) increased the antioxidant activity of the roasted Se-coffee, although lower initial antioxidant capacity was observed in the green Se-coffee. The participation of selenomethionine in the Maillard reaction during roasting enhances the formation of organoselenium of different structures and reactivity which can contribute to the increase in antioxidant activity of the Se-coffee [40,41].

**Figure 3:**
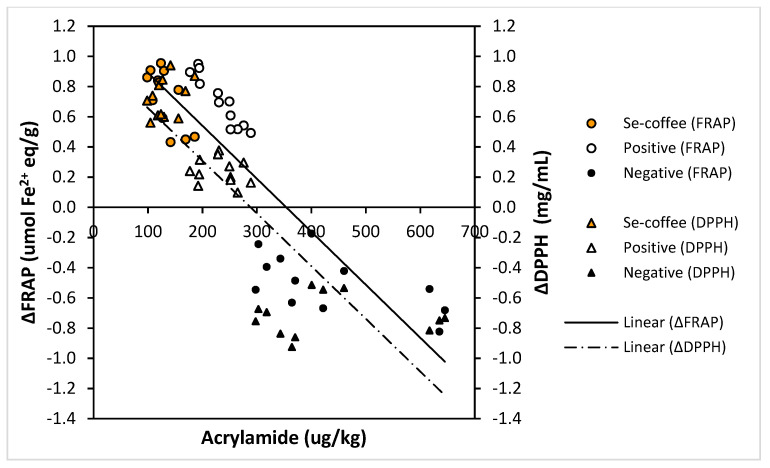
Correlation between acrylamide level and change of antioxidant capacities of Se-coffee (pretreated with selenium), positive control (pretreated without selenium), and negative control (untreated) measured from the difference of FRAP values (∆FRAP) and DPPH EC_50_ values (∆DPPH) between green and roasted coffees.

The change of antioxidant capacity of coffee beans after roasting measured from the difference of FRAP values (∆FRAP) and DPPH EC_50_ values (∆DPPH) between green and roasted coffees showed a strong correlation with acrylamide levels of the roasted coffee as shown in Figure 3. The acrylamide reduction increased with increase in positive values of the ∆FRAP and ∆DPPH with significant Pearson’s correlation of −0.858 (*p* < 0.05) and −0.836 (*p* < 0.05), respectively. These results suggest that high antioxidant capacity of Se-coffee attributed to the presence of organoselenium may acted as an inhibitor to the formation of acrylamide. Cheng et al. (2015) reported similar observations that the reduction of acrylamide levels in a Maillard reaction model strongly correlated with the addition of flavonoids at a moderate level without significantly contributing to the final antioxidant capacity of the Millard reaction products [56]. Moreover, the reduction in the selenium content of the roasted Se-coffee in comparison to the green Se-coffee beans (Figure 1) suggests the participation of selenomethionine in the Maillard in mitigating acrylamide in the Se-coffee.

On the other hand, the negative values of the ∆FRAP and ∆DPPH represented by the negative control samples (untreated coffee beans) strongly correlated with the increase in acrylamide levels. High concentration of antioxidants with carbonyl compounds (predominantly chlorogenic acids) in the green negative control can trigger the decomposition of sugars and further reaction with Maillard reaction intermediates such as 3-aminopropionamide to promote the formation of acrylamide [14,56,57]. Pretreatment of the green beans may suggest lower contribution of phenolic compounds in the formation of α-dicarbonyl compounds from sugars and carbohydrates present in the green coffee beans that lead to lower acrylamide formation compared to the negative control.

Two-way ANOVA analysis showed that roasting method has a larger contribution to the reducing activity of the roasted coffee compared to the selenium pretreatment with significant interaction effect (Table 2). However, their radical scavenging activity was greatly influenced by selenium pretreatment with significant interaction effect. Compared to the conventional roasting, the superheated steam roasting significantly increased antioxidant activity of the negative control samples. The thermal degradation of phenolic antioxidants, predominantly chlorogenic acids and their degradation products (caffeic, ferulic, and coumaric acids) during roasting were reported at a lower degree under oxygen-free condition of superheated steam roasting compared to the conventional roasting [42,55].

### 3.4. Effects of Selenium Pretreatment on Color of Roasted coffee

**Figure 4:**
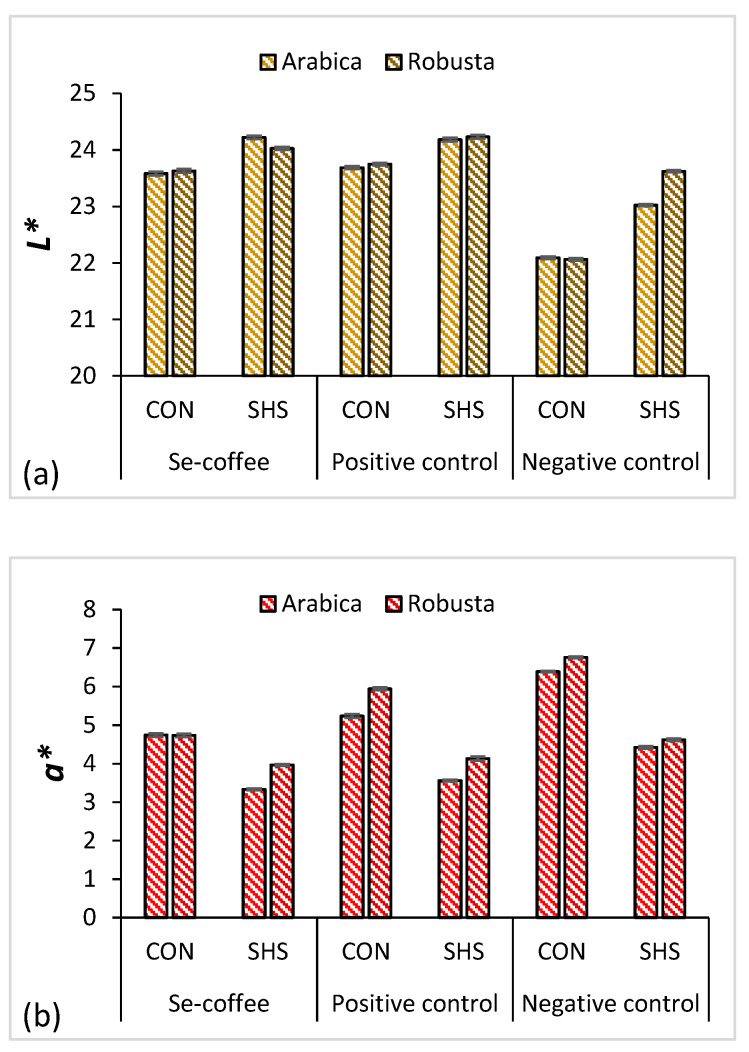

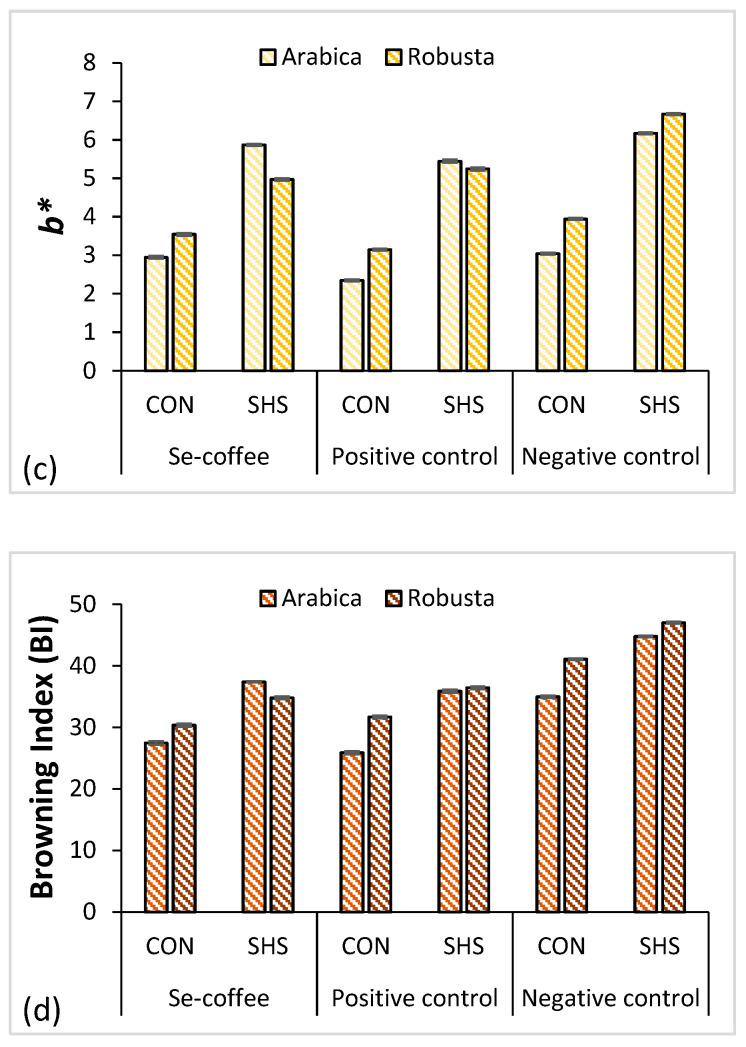
Chromatic parameters (**a**) L*, (**b**) a*, (**c**) b*, and (**d**) browning index (BI) of Arabica and Robusta ground Se-coffee (pretreated with selenium), positive control (pretreated without selenium), and negative control (untreated) roasted via conventional (CON) and superheated steam (SHS) roasting.

Color of roasted coffee develops from Maillard reaction, caramelization, and polyphenols oxidation, also determines degree of roasting of the coffee beans. The roasting degree of coffee has been proposed by Sacchetti et al. (2009) from the L* value as follow: Light roasted (L* > 35), medium roasted (25 < L* < 35), and dark roasted (L* < 25) [54]. The effects of pretreatment and different roasting methods on the formation of browning pigments during coffee roasting were assessed using browning index (BI) and color parameters (L*, a*, and b*), as shown in Figure 4. All samples were categorized as dark roast with L* values lower than 25. The L* values of Se-coffee (23.6–24.2) and positive control (23.7–24.2) were higher compared to the negative control (22.1–23.6) inferring formation of lighter coffee beans in the pretreated beans. The formation of brown color pigments from sugar caramelization and Maillard reaction can be impacted by the pretreatments due to the removal of soluble sugar components during soaking. The two-way ANOVA analysis of color and BI obtained significant main effects of both pretreatment and roasting methods and significant interaction effect with stronger influence of pretreatment on L* and roasting method on BI.

The Se-coffee showed lower a* value compared to the positive and negative controls, in agreement with the correlation between acrylamide and a* value in roasted coffee beans [58]. The pretreatments also reduced b* value and BI which can be associated with lower Maillard reaction products. Although Se-coffee showed lower L*, a*, and b* values compared to the positive control, it has higher BI signifying preferred overall characteristic of the brown color of roasted coffee beans. On the other hand, the superheated steam roasting increased L*, b*, BI, and decreased a* value of the SHS samples compared to the conventional roasted samples which associated with the ability of superheated steam to maintain the melanoidin and phenolic compounds from thermal degradation [9].

## 4. Conclusions

Organic selenium as antioxidant additive supplemented via pretreatment of green coffee beans was effective in reducing acrylamide formation by 73% to a level 70% below the benchmark levels established by the European Commission for roasted coffee (400 μg/kg). Although pretreatments of green coffee reduced the antioxidant activity of green beans, roasting resulted in a comparable antioxidant activity between samples of different pretreatments. The increase in antioxidation capacity from selenium fortification and removal of water-soluble precursors of the Maillard reaction may explain the acrylamide reduction mechanism of Se-coffee. Superheated steam roasting significantly reduced acrylamide levels up to 32% and increased antioxidant activity which was only noticed in the untreated coffee beans. This study suggests that the superheated steam roasting is an effective physical method in mitigating acrylamide in coffee. However, the chemical pretreatment of selenium supplementation is more effective attributing to the removal of associated precursors and an increase in antioxidation activity which also provides supplementation of selenium to coffee beverages. Besides costing, sensory properties of the Se-coffee remain the most important consumer parameter warrants study.

## Figures and Tables

**Table 1:** Ferric reducing antioxidant power (FRAP) reducing activity and 2,2-diphenyl-1-picrylhydrazyl (DPPH) scavenging activity expressed as EC_50_ for green Se-coffee (pretreated with selenium), positive control (pretreated without selenium), and negative control (untreated).

Pretreatments	Arabica Coffee		Robusta Coffee	
	FRAP ^1^ (mmol Fe^2+^ eq/g)	DPPH EC_50_^1^ (mg/mL)	FRAP ^1^ (mmol Fe^2+^ eq/g)	DPPH EC_50_^1^ (mg/mL)
Se-Coffee	0.58 ± 0.02 ^a^	3.19 ± 0.02 ^b^	1.00 ± 0.03 ^b^	3.20 ± 0.02 ^b^
Positive Control	0.74 ± 0.01 ^a^	3.38 ± 0.02 ^c^	0.84 ± 0.02 ^a^	3.15 ± 0.05 ^b^
Negative Control	1.86 ± 0.07 ^b^	1.93 ± 0.01 ^a^	2.07 ± 0.02 ^c^	1.92 ± 0.03 ^a^

^1^ Values are mean (n = 3) ± s.d. Mean values within pretreatment followed by different uppercase letter are significantly different at *p* < 0.05.

**Table 2:** FRAP reducing activity and DPPH scavenging activity (expressed as EC_50_) of Se-coffee, positive control and negative control roasted via conventional (CON) and superheated steam (SHS) roasting.

	Arabica Coffee		Robusta Coffee	
	FRAP ^1^ (mmol Fe^2+^ eq/g)	DPPH EC_50_^1^ (mg/mL)	FRAP ^1^ (mmol Fe^2+^ eq/g)	DPPH EC_50_^1^ (mg/mL)
Se-coffee				
CON	1.42 ± 0.01 ^Ba^	2.59 ± 0.03 ^Aa^	1.45 ± 0.05 ^Ba^	2.33 ± 0.08 ^Aa^
SHS	1.49 ± 0.01 ^Bb^	2.57 ± 0.03 ^Aa^	1.71 ±0.03 ^Bb^	2.30 ± 0.05 ^Aa^
Positive control				
CON	1.23 ± 0.03 ^Aa^	3.03 ± 0.02 ^Ca^	1.36 ± 0.04 ^Aa^	2.96 ± 0.05 ^Ca^
SHS	1.40 ± 0.04 ^Ab^	3.18 ± 0.04 ^Bb^	1.45 ±0.07 ^Ab^	2.93 ± 0.01 ^Ba^
Negative control				
CON	1.37 ± 0.02 ^Ba^	2.80 ± 0.04 ^Bb^	1.39 ± 0.01 ^Aa^	2.69 ± 0.04 ^Bb^
SHS	1.46 ± 0.02 ^Bb^	2.64 ± 0.03 ^Aa^	1.65 ±0.03 ^Bb^	2.45 ± 0.04 ^Aa^
Two-way ANOVA analysis (*F*-value)				
Pretreatment	34.8 ***	295.5 ***	15.8 ***	215.5 ***
Roasting	59.4 ***	NS	62.4 ***	8.6 ***
Pretreatment × Roasting	4.6 ***	24.9 ***	4.8 ***	14.2 ***

^1^ Values are mean (n = 3) ± s.d. Mean values within pretreatment followed by different uppercase letter are significantly different at *p* < 0.05. Mean values within roasting methods followed by different lowercase letter are significantly different at *p* < 0.05. *** Significance at *p* < 0.05; NS, non-significant.
